# Altered expression and localization of nuclear envelope proteins in a prostate cancer cell system

**DOI:** 10.1007/s11033-024-09836-4

**Published:** 2024-08-08

**Authors:** Ariana Sandoval, Efrain Garrido, Javier Camacho, Jonathan Javier Magaña, Bulmaro Cisneros

**Affiliations:** 1 Departamento de Genética y Biología Molecular, Centro de Investigación y de Estudios Avanzados del Instituto Politécnico Nacional (CINVESTAV), Ciudad de México, 07360 México; 2 Departamento de Farmacología, Centro de Investigación y de Estudios Avanzados (CINVESTAV), Ciudad de México, 07360 México; 3 Laboratorio de Medicina Genómica, Departamento de Genética (CENIAQ), Instituto Nacional de Rehabilitación-Luis Guillermo Ibarra Ibarra (INR-LGII), Ciudad de México, 14389 México; 4 Departamento de Bioingeniería, Escuela de Ingeniería y Ciencias, Tecnologico de Monterrey, Campus Ciudad de México, 14380 México

**Keywords:** Prostate cancer, Nuclear envelope, Nuclear lamins, Emerin, β-DG, Cell invasiveness.

## Abstract

**Background:**

The nuclear envelope (NE), which is composed of the outer and inner nuclear membranes, the nuclear pore complex and the nuclear lamina, regulates a plethora of cellular processes, including those that restrict cancer development (genomic stability, cell cycle regulation, and cell migration). Thus, impaired NE is functionally related to tumorigenesis, and monitoring of NE alterations is used to diagnose cancer. However, the chronology of NE changes occurring during cancer evolution and the connection between them remained to be precisely defined, due to the lack of appropriate cell models.

**Methods:**

The expression and subcellular localization of NE proteins (lamins A/C and B1 and the inner nuclear membrane proteins emerin and β-dystroglycan [β-DG]) during prostate cancer progression were analyzed, using confocal microscopy and western blot assays, and a prostate cancer cell system comprising RWPE-1 epithelial prostate cells and several prostate cancer cell lines with different invasiveness.

**Results:**

Deformed nuclei and the mislocalization and low expression of lamin A/C, lamin B1, and emerin became more prominent as the invasiveness of the prostate cancer lines increased. Suppression of lamin A/C expression was an early event during prostate cancer evolution, while a more extensive deregulation of NE proteins, including β-DG, occurred in metastatic prostate cells.

**Conclusions:**

The RWPE-1 cell line-based system was found to be suitable for the correlation of NE impairment with prostate cancer invasiveness and determination of the chronology of NE alterations during prostate carcinogenesis. Further study of this cell system would help to identify biomarkers for prostate cancer prognosis and diagnosis.

**Supplementary Information:**

The online version contains supplementary material available at 10.1007/s11033-024-09836-4.

## Introduction

The nuclear envelope (NE) is a highly regulated membrane barrier that preserves the genome and separates physically and functionally the nucleus from the cytoplasm in metazoan [1]. The NE comprises the inner and outer nuclear membranes, the nuclear pore complex (NPC), and the nuclear lamina [2]. It also contains transmembrane and peripherally associated proteins [3]. The nuclear lamina is a dense fibrillar meshwork situated beneath the inner nuclear membrane and consists of intermediate filaments, namely type A (A and C) and type B (B1 and B2) lamins [4, 5]. The nuclear lamina connects NE proteins to genomic DNA and chromatin, which together act as platform for a variety of cellular processes, including chromatin organization, gene expression, DNA repair, cell cycle regulation and mechanotransduction [4, 6]. The NE controls several cellular processes whose dysregulation is observed in cancer, including genomic stability cell migration and resistance to mechanical stress [7]. Thus, it is not surprising that altered expression of NE components is prevalent in multiple cancer types [8–10]. Deregulation of NE proteins results in abnormal nuclei with enlarged size and aberrant shape, a hallmark of cancer cells commonly used for diagnosis [11]. Then, since NE dysfunction is functionality related to tumorigenesis, the design of treatments using NE proteins as therapeutic targets is a promising avenue to counteract cancer [12, 13].

Prostate cancer is the most frequently diagnosed cancer in men worldwide, followed by lung cancer (World Health Organization, 2020) [14]. It is an age-related disease influenced by genetic and environmental factors [15]. Prostate cancer is determined by the malignant transformation of prostate cells through a multistep process known as epithelial-to- mesenchymal transition. Once the primary tumor is established, it progresses by locally invading the prostate, and further spread of cancer cells to other tissues (metastasis) culminates in the death of the patient [16]. The FDA-approved tools for prostate cancer detection include prostate-specific antigen (PSA) blood test, histologic analysis of prostate biopsy using Gleason score, imaging and ultrasound tests, and an expression array of group of cell cycle, DNA repair and breast cancer (BRCA) genes [17]. However, accurate and early diagnosis of prostate cancer that avoids overdiagnosis/overtreatment of patients is still a necessity.

Monitoring of nuclear morphology and expression of NE proteins has also been applied to both prognosis and diagnosis of prostate cancer [18–20]. In particular, dysregulation of NE proteins including nuclear lamina proteins and inner nuclear membrane proteins has been observed in both clinical prostate cancer samples and prostate cancer cell lines [19, 21–23]. However, the simultaneous analysis of NE proteins in a controlled prostate cancer cell model to determine the chronology of events leading to NE dysfunction and the association of NE alterations with cell invasiveness remains to be addressed.

In this study, we analyzed the expression and subcellular localization of a number of NE proteins previously implicated in prostate cancer development, namely lamin A/C and lamin B1, and the inner nuclear membrane proteins emerin and β-dystroglycan (β-DG), using a cellular system consisting of the prostate epithelial cell line RWPE-1, and four prostate cancer cell lines with different metastatic potential WPE1-NA22, WPE1-NB14, WPE1-NB26 and DU145 [24].

## Materials and methods

### Cell culture

The prostate cell lines were acquired from American Type Culture Collection (ATCC): RWPE-1 (non-tumorigenic human prostate epithelial cell line; CRL-3607) and its derived tumorigenic prostate cancer cell lines WPE1-NA22 (CRL-2849), WPE1-NB14 (CRL-2850) and WPE1-NB26 (CRL-2852) [24]. These cell lines were grown in complete Keratinocyte serum free medium (KSFM) medium (Sigma, Saint Louis, MOI) containing 50 µg/ml bovine pituitary extract, 5 ng/ml human EGF and 1% antibiotic/antimycotic mixture (penicillin 100 U/ml, streptomycin 100 µg/ml, and Fungizone 0.25 µg/ml) (Sigma, Saint Louis, MOI). The human prostate metastatic cell line DU145 [24], acquired from ATCC (HTB-81), was maintained in RPMI-1640 medium supplemented with 5% fetal bovine serum (FBS) (Invitrogen, Carlsbad, CA). All cell lines were maintained in a humidified 5% CO2 atmosphere at 37◌֯C.

### Antibodies

The following primary antibodies were used. Rabbit polyclonal anti-lamin B1 (ab16048) and mouse monoclonal anti-lamin A/C (ab8984) antibodies (Abcam, Cambridge, UK). Rabbit polyclonal anti-lamin A/C (sc-20681) and anti-emerin (sc-15378) antibodies (Santa Cruz Biotechnology, CA, USA). The mouse monoclonal anti-β-DG antibody (MANDAG2) was previously reported [25], while the mouse monoclonal anti-β-actin antibody was a gift from Dr. Manuel Hernández (CINVESTAV, Mexico City).

### Immunofluorescence and confocal microscopy analysis

Cells seeded on coverslips to 65% confluence were fixed with 4% paraformaldehyde in PBS for 10 min, permeabilized with 0.2% Triton X-100 in PBS for 10 min, blocked with 1% fetal FBS and 0.5% gelatin in PBS for 20 min and incubated overnight in a humid chamber at 4^0^C with the appropriate primary antibodies (anti-lamin A/C at 1:250 dilution; anti-lamin B1 at 1:300 dilution; emerin at 1:100 dilution; and anti-β-DG at 1:25 dilution). The next day, cells were washed twice with PBS for 5 min and then incubated with the appropriate fluorochrome-conjugated secondary antibodies (anti-mouse at 1:50 dilution and anti-rabbit at 1:50 dilution; Jackson Immunoresearch Laboratories, West Grove, PA) for 1 h at room temperature. Finally, coverslip preparations were incubated with 0.2 µg/mL diamidino-2-phenylindole (DAPI; Sigma-Aldrich, St. Louis, MO, USA) for 10 min at room temperature to visualize nuclei, mounted on microscope slides with VectaShield (Vector Laboratories Inc. Burlingame, CA, USA) and further visualized with an Eclipse Ti-E inverted confocal laser scanning microscope (Nikon, Japan). Images were analyzed using Image J1.62 software (NIH).

### Western blotting

Aliquots of cell lysates (50 µg) were electrophoresed on 10% SDS-polyacrylamide gels and transferred onto nitrocellulose membranes (Hybond-Nþ, Amersham Pharmacia, GE Healthcare, Buckinghamshire, UK) at 35 V for 60 min using a Transblot apparatus (Bio-Rad, Hercules, CA, USA). The membranes were blocked in TBST [100 mM Tris-HCl pH 8.0, 150mM NaCl, 0.5% (v/v) Tween-20] with 15% low- fat dried milk for 1 h at room temperature, and then incubated overnight at 4 °C with the appropriate primary antibodies diluted in 3% low-fat dried milk (anti-lamin A/C at 1:4,000 dilution; anti-lamin B1 at 1:1,000 dilution; emerin at 1:1,000 dilution; anti-β-DG at 1:500 dilution; and acti-actin at 1:1,000 dilution). The following day, the specific protein signal was developed using the appropriate Horseradish peroxidase-conjugated secondary antibodies (anti-mouse at 1:10,000 dilution and anti-rabbit at 1:20,000 dilution; Amersham Pharmacia, GE Healthcare, Buckinghamshire, UK), and the enhanced chemiluminescence western blotting detection system (Amersham Pharmacia, GE Healthcare). Radiation was detected after 3–5 min of exposure to an X-ray film in the dark, except for the blots for β-DG, which required 15 min of exposure.

### Statistical analysis

Data from three independent experiments for each assay in this study were analyzed by unpaired Student´s t-test, using GraphPad Prism 6 statistical analysis software.

## Results

### Mislocalization and altered levels of lamins A/C and B1 during prostate cancer progression

**Fig. 1:**
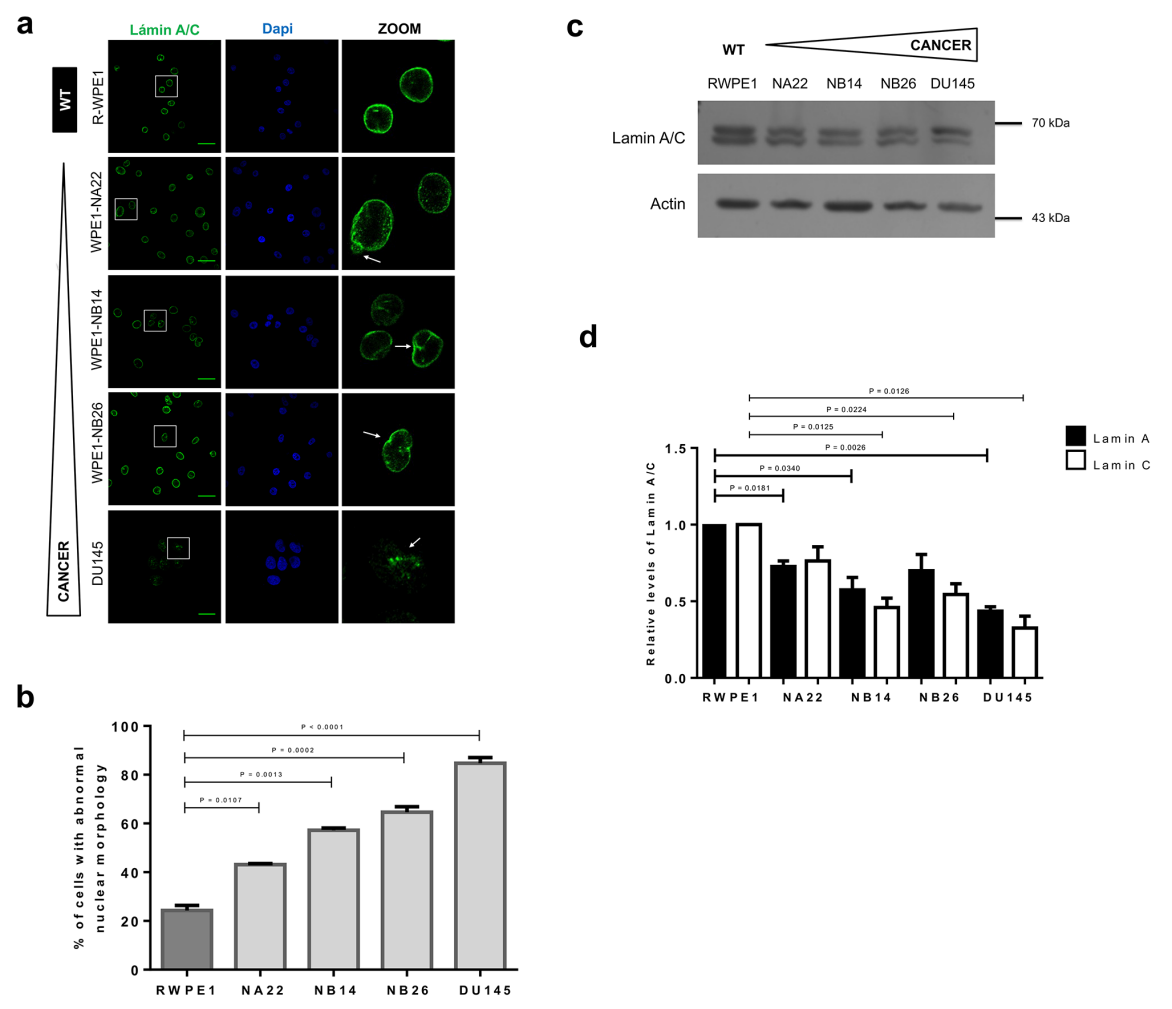
Abnormal localization and protein expression of lamin A/C in prostate cancer cell lines. (**a**) The indicated prostate and prostate cancer cell lines were grown on coverslips, fixed and immunostained for lamin A/C. Nuclei were stained with DAPI prior to analysis by confocal laser scanning microscopy (CLSM), and typical images are shown. Scale bar, 33 μm for WPE1 cells and 22 μm for DU145 cell line. Arrows denote nuclear deformities. (**b**) The percentage of cells with aberrant nuclei was calculated from three independent experiments (*n* = 50 nuclei), with p values indicating significant differences (unpaired t test). Nuclei were considered normal if they were spheroid or ellipsoid and abnormal if they were blebbed, lobulated or contained invaginations. (**c**) Cell lysates were subjected to Western blot analysis using antibodies specific for lamina A/C and actin (loading control); typical blots from three independent experiments are shown. (**d**) Densitometric analysis of immunoblot autoradiograms was performed to estimate lamin A/C protein expression. Relative lamin A/C protein levels from control cells (RWPE1) were set to 1 for comparison. Data represent the mean ± SD of three separate experiments, with significant differences determined by unpaired t test

**Fig. 2:**
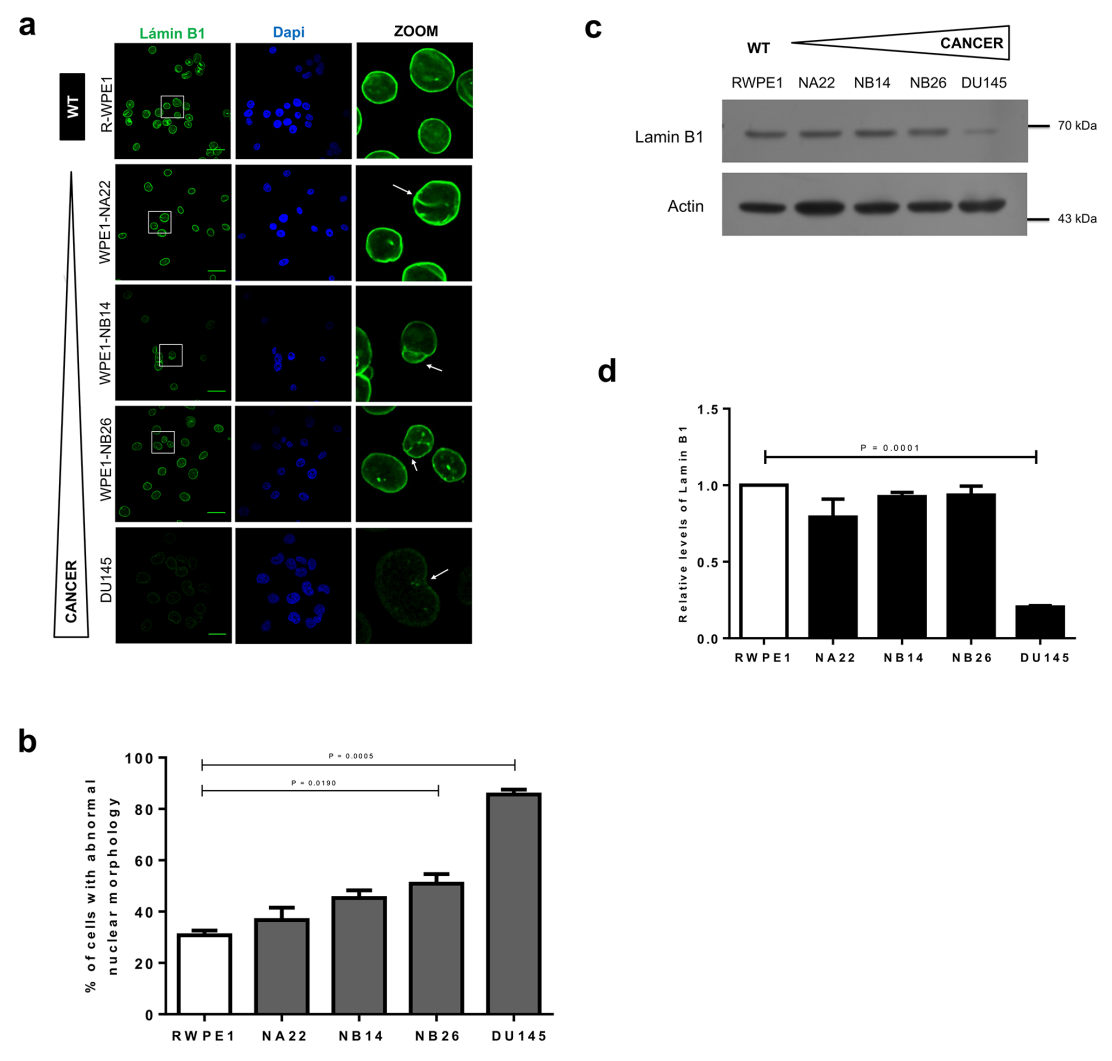
Distribution and protein levels of lamin B1 in the prostate cancer cell lines. (**a**) The prostate and prostate cancer cell lines were seeded on coverslips, immunolabeled for lamin B1 and counterstained with DAPI to decorate the nuclei. Cell preparations were visualized by CLSM and representative images are shown. Scale bar, 33 μm for WPE1 cells and 22 μm for DU145 cell line. Arrows denote nuclear deformities. (**b**) The percentage of distorted nuclei was evaluated from three separate experiments (*n* = 50 nuclei), with p values indicating significant differences (unpaired t test). Nuclei were classified as in Fig. 1. (**c**) Lysates were analyzed by immunoblotting with primary antibodies against lamina B1 and actin (loading control); representative blots from three separate experiments are shown. (**d**) Immunoblots were subjected to densitometric analysis to quantify the amount of lamin B1. The relative expression of lamin B1 from control cells (RWPE1) was set to 1 for comparison. Data represent the mean ± SD from three separate experiments, with significant differences determined by unpaired t test

We analyzed the content and subcellular localization of different NE proteins during prostate carcinogenesis, using a human cancer cell system composed of different prostate cancer cell lines [24, 26], which together recreate the different stages of prostate cancer cell progression/invasiveness. First, we examined the distribution of lamins A/C and B1 in the different prostate cell lines, namely WPE1 (normal prostate epithelial cells), WPE1-NA22, WPE1-NB14 and WPE1-NB26 (WPE1-derived prostate cancer cell lines with increasing grades of invasiveness), and DU145 (metastatic prostate cancer cell line) by confocal laser scanning microscopy (CLSM). The typical ring-like immunostaining pattern with some nucleoplasmic foci, was observed for lamin A/C and lamin B1 in all prostate cell lines analyzed; however, a marked decrease in the fluorescence intensity of lamins was specifically observed in the metastatic cell line DU145 (Figs. 1A and 2A). Furthermore, nuclear deformities including invaginations and blebs, were evident in the prostate cancer cell lines immunolabeled for lamin A/C and lamin B1, with the metastatic DU145 cells showing the highest abundance of aberrant nuclei (Figs. 1A-B and 2A-B). Consistent with these results the levels of lamin A and lamin C were found to be significantly decreased in prostate cancer cell Iines, compared with R-WPE1 control cells (Fig. 1C and D), with DU145 cells having the lowest content of lamin A/C. With respect to lamin B1, its content was found to be reduced exclusively in DU145 cells (Fig. 2C and D).

### The distribution and levels of the NE proteins emerin and β-DG is impaired in the metastatic prostate cancer cells DU145

**Fig. 3:**
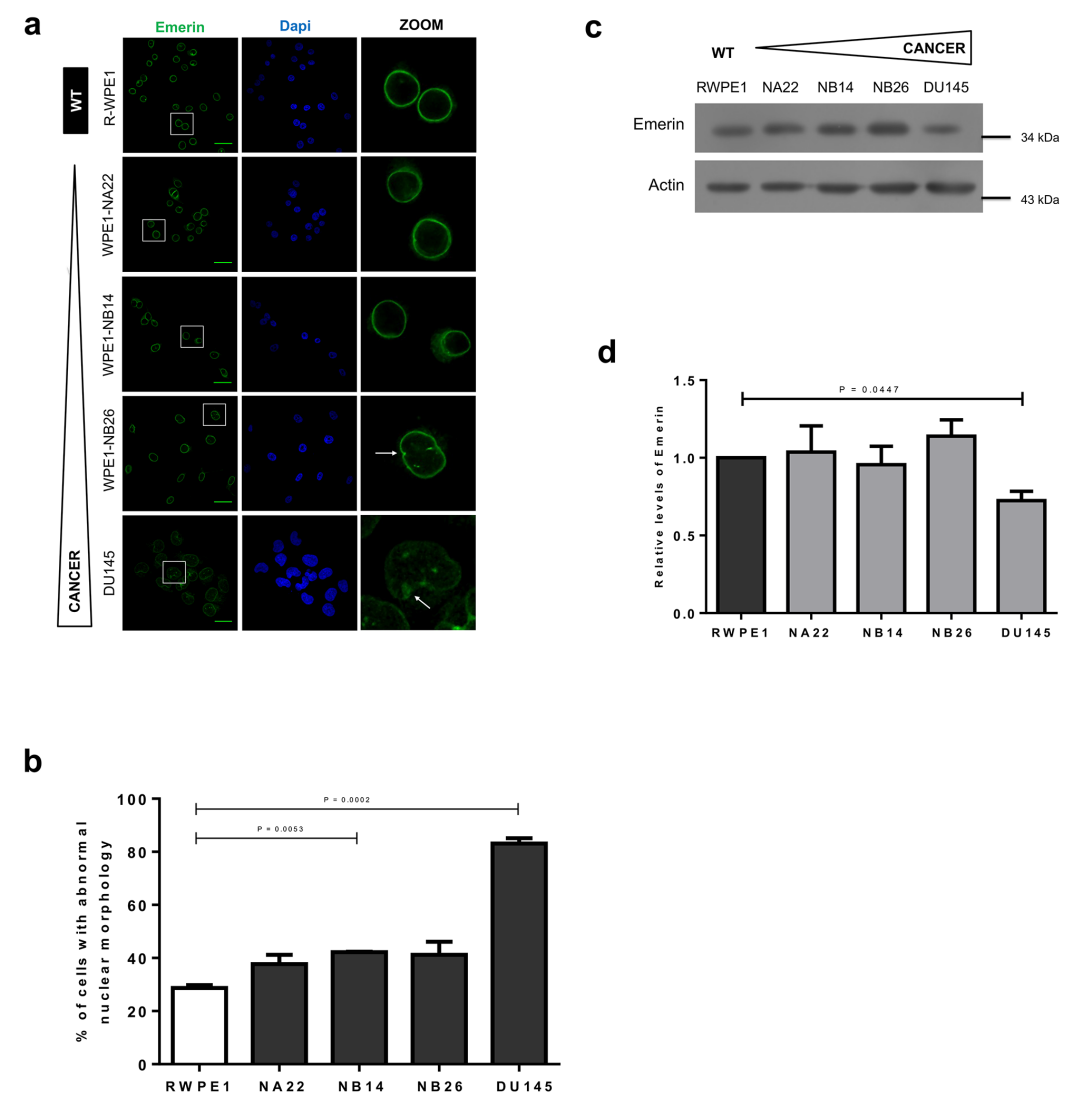
Localization and protein expression of emerin during prostate cancer progression. (**a**) Prostate and cancerous prostate cells grown on coverslips were fixed and immunostained for emerin, then nuclei were labeled with DAPI before CLSM). Typical images are shown. Scale bar, 33 μm for WPE1 cells and 22 μm for DU145 cell line. Arrows denote nuclear deformities. (**b**) The percentage of cells with aberrant nuclei was calculated from three independent experiments (*n* = 50 nuclei), with p values indicating significant 11 differences (unpaired t test). Nuclei were scored as in Fig. 1. (**c**) Lysates from the different cell lines were analyzed by Western blotting using antibodies specific for lamina A/C and actin (loading control), with representative blots from three independent experiments shown. (**d**) The relative protein level of emerin was calculated and the value from control cells (RWPE1) was set to 1 for comparison. Data represent the mean ± SD of three separate experiments, with significant differences determined by unpaired t test

**Fig. 4:**
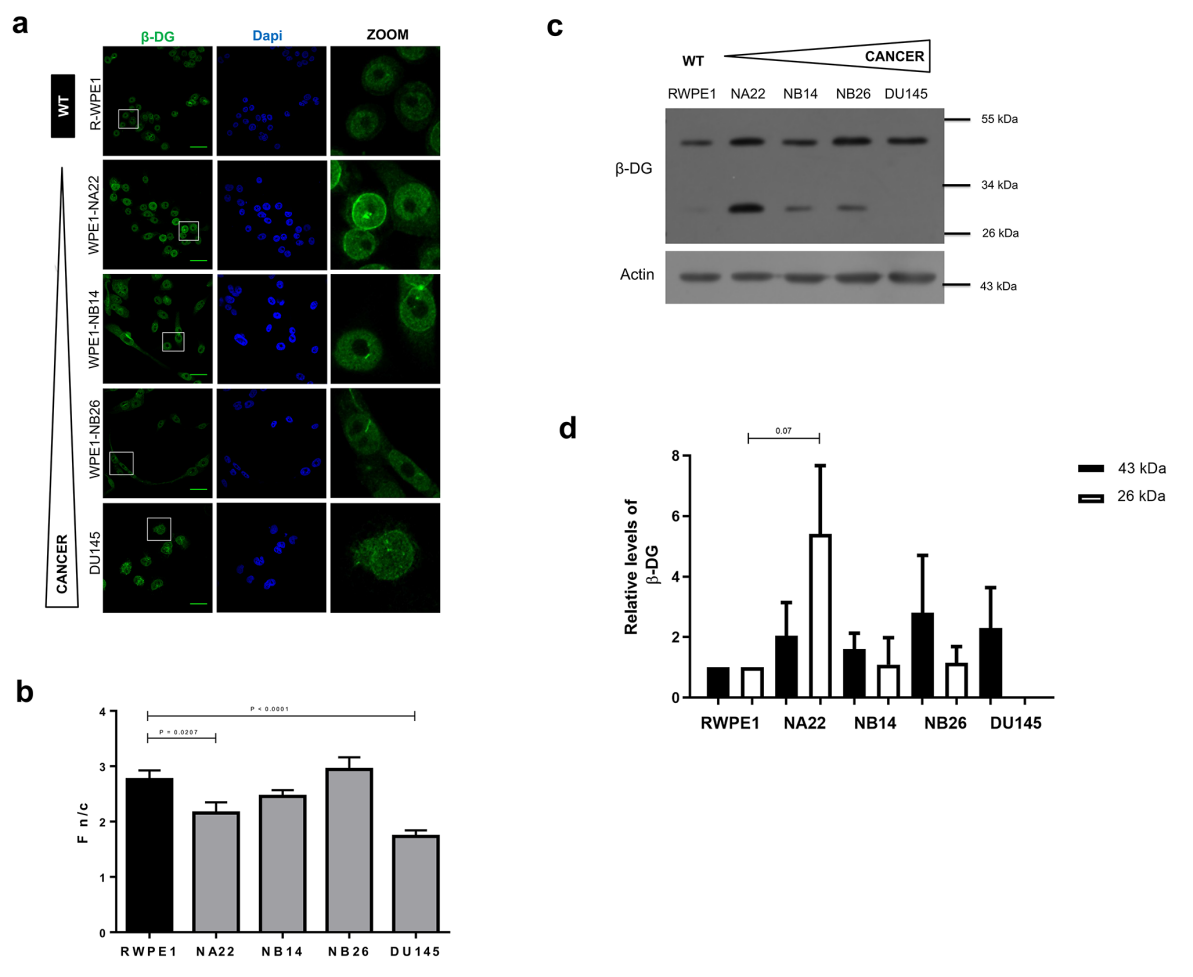
Analysis of β-dystroglycan during prostate cancer progression. (**a**) Cells were seeded on coverslips prior to immunolabeling for β-DG, counterstained with DAPI, and further subjected to CLSM. Typical images are shown. Scale bar, 33 μm for WPE1 cells and DU145 cell line. (**b**) The nuclear-cytoplasmic ratio of β-DG immunofluorescence intensity (F n/c) was calculated from three independent experiments (*n* = 100 nuclei); p values indicate significant differences (unpaired t- test). (**c**) Cell lysates were fractionated by SDS-PAGE and further examined by Western blotting using antibodies against β-DG and actin (loading control), with representative blots from three separate assays shown. (**d**) The relative levels of full-length β-DG (43 kDa) and its proteolytic fragment (26 kDa) were calculated and the value obtained from control cells (RWPE1) was set to 1 for comparison. Data represent the mean ± SD of three separate experiments, with significant differences indicated by unpaired t- test

To extend the analysis of NE proteins during prostate carcinogenesis, we evaluated the subcellular localization and protein expression of the inner nuclear membrane proteins emerin and β-DG, which are also nuclear lamina-associated proteins. A clear emerin labeling at the nuclear periphery was observed in all prostate cell lines examined, with the presence of deformed nuclei in both NB14 and DU145 cells, compared to RWPE1 control cells (Fig. 3A-B). Interestingly, a significant decrease of emerin was detected in DU145 metastatic cells by CLSM and Western blot assays (Fig. 3A, C-D). On the other hand, β-DG showed a predominant nuclear localization with a weak signal in the cytoplasm in all prostate cell lines (Fig. 4A). Notably, nuclear labeling of β-DG was excluded from some nuclear regions, which must correspond to the position of nucleoli, in all prostate cell lines, except for DU145 cells, where labeling of β-DG was extended throughout the nucleus in most cells (Fig. 4A). The nuclear/cytoplasmic fluorescence intensity (F n/c) was significantly lower in NA22 and DUA 145 cells (Fig. 4B). Immunoblotting analysis for β-DG revealed the presence of 43 kDa and 26 kDa fragments, which must correspond to full-length β-DG and its proteolytically cleaved intracellular domain respectively [27, 28]. The 26 kDa proteolytic fragment of β-DG was present in all prostate cell lines, except DU145 cells, with NA22 cells having the highest content of this protein form (Fig. 4C-D).

## Discussion

In this study, we used a well-established prostate cancer cell system to analyze NE integrity during prostate cancer development by evaluating the content and subcellular localization of different NE proteins. The prostate cancer cell system consists of the prostate cell line RWPE-1 (control) and its derived prostate cancer cell lines WPE1-NA22 (9% invasiveness), WPE1-NB14 (30% invasiveness) and WPE1-NB26 (95% invasiveness), which were obtained by exposure to increasing concentrations of N-methyl-N-nitrosourea (MNU). The metastatic DU145 cell line, considered to have 100% invasiveness [24], completed the spectrum of prostate cancer cell lines analyzed. We choose the lamins A/C and B1, and the inner nuclear membrane proteins emerin and β-DG for this study because, they are involved in several cellular processes commonly deregulated in cancer, including cell signaling, chromatin organization, gene expression, nuclear structure and function and mechano- transduction [6]; and because they have previously been used as biomarkers of prostate cancer evolution [29]. We found that the percentage of cells with distorted nuclei as well as the decrease in lamin A/C levels were higher as the invasiveness of the prostate cancer cell lines increased, with the metastatic cell line (DU145) showing mislocalization and decreased expression of either lamin A/C, lamin B1, and emerin. With respect to β-DG, the nucleocytoplasmic fluorescence ratio of this protein was found to decrease in NA22 and DU145 cells; in addition, the 26 kDa proteolytic fragment of β-DG was absent exclusively in DU145 cells. Collectively our results suggest that the expression and proper localization of nuclear lamina and inner nuclear membrane proteins are necessary for nuclear integrity, and that disruption of the NE multiprotein system appears to promote prostate cancer development. Interestingly, downregulation of lamin A/C appears to be an early event in prostate carcinogenesis, while impairment of the remaining NE proteins analyzed is a later step in prostate cancer evolution and is a hallmark of metastatic prostate cells.

Previous studies reported an increase of lamin A/C in prostate cancer tissue, which is thought to promote prostate cancer cell growth, migration and invasion via PI3K/AKT/PTEN signaling [22]. In contrast, and in support of our data, decreased lamin A/C protein expression was observed in several metastatic prostate cancer cell lines, which in turn caused nuclear stiffness [21]. Similarly, forced downregulation of lamin A/C expression in metastatic prostate cells resulted in nuclear membrane blebbing and enhanced migration and invasion [20]. On the other hand, lamin B1 showed reduced protein levels in the metastatic prostate cancer cell line LNCaP, compared to the control prostate cell line RWPE-1 [21]. With respect to emerin, knockdown of emerin in the prostate cancer cell lines DU145, PC3 and LNCaP resulted in altered nuclear morphology and increased migration and invasiveness [20]. Furthermore, intracardiac injection of emerin-depleted DU145 cells into severe combined immunodeficient (SCID) mice induced metastases in a variety of organs, compared to control DU145 cells [20]. The prognostic value of lamins in clinical samples has also been addressed. In a tissue microarray of prostate cancer patients, decreased levels of lamin A/C and B2 were found to correlate with increased risk of lymph node metastasis and disease-specific death, while increased levels of lamin B1 were associated with disease recurrence [19]. Regarding β-DG, there is increasing evidence that this protein plays a role in prostate cancer [25, 28, 30]. Experimental manipulation of β-DG expression in metastatic LNCaP prostate cells showed that loss of β-DG facilitated cell growth in an anchorage- independent manner, whereas overexpression of β-DG favored Matrigel invasion [28]. Furthermore, a progressive loss of β-DG immunoreactivity on prostate epithelia was found to correlate with increasing Gleason grade in tumor tissue samples [28]. Nuclear function of β-DG may contribute to prostate cancer; because nuclear import of tyrosine-phosphorylated β-DG results in activation of the oncogenic transcription factor ETS variant 1 (ETV1) [25]. Considering this background and our data, we hypothesized that early suppression of lamin A/C expression in prostate cells affects NE structure, with lamin B1 losing its stability consequently. In parallel, lamin A/C deficiency could render emerin susceptible to proteasomal degradation, as previously described [31]. In addition, decreased nuclear localization of β-DG found in DU145 metastatic prostate cells may contribute to NE impairment, as this protein is involved in the stability of lamin B1 and emerin [32, 33]. Lamin B1 deficiency could in turn disrupt the interplay of the nuclear lamina with both inner nuclear membrane proteins and chromatin, as lamin B1 has the ability to bind DNA through lamina- associated heterochromatin domains (termed LAD) and indirectly associate with chromatin via LEM proteins [LAP2 (lamina-associated polypeptide 2)-emerin-MAN1] [34]. Finally, as the β-DG 26 kDa intracellular proteolytic fragment has been implicated in the maintenance of nucleoli morphology [35], its absence in the metastatic DU145 cells may contribute to nucleolar expansion, a well-recognized hallmark of cancer cells [36, 37].

Although prostate cancer cell lines are helpful to determine the mechanisms underlying tumorigenesis, they have some limitations. These cell lines are selected from specific tumor subsets and solely grown on 2-dimensional monolayer, lacking the interactions stablished between the cellular and extracellular environments, and consequently losing the heterogeneity observed in primary prostate cancer cells. To overcome these limitations 3- dimensional cultures of patient-derived xenografts, organoid and spheroid have been developed, which properly recapitulate the cellular diversity and histology features of patient prostate tumors, retaining then, structural genomic events, epigenetic features, and gene expression programs [38, 39].

## Conclusion

In summary, we demonstrate the usefulness of the RWPE-1 cell line-based system to correlate NE impairment with the invasive capacity of prostate cancer cells and provide evidence for the chronology of NE alterations occurring during cancer progression, with downregulation of lamina A/C being an early event that can be used for diagnosis in prostate cancer onset.

## Electronic supplementary material

Below is the link to the electronic supplementary material.

Supplementary Material 1

## Data Availability

Data is provided within the manuscript file.

## Abbreviations

NE: Nuclear envelope
CLSM: Confocal laser scanning microscopy
LEM proteins: LAP2 (lamina-associated polypeptide 2)-emerin-MAN1
FDA: Federal Drug Administration
PSA: Prostate Specific Antigen
MNU: N-methyl-N-nitrosourea
EGF: Epidermal growth factor
PBS: Phosphate buffered saline
