# Supplementary material for: Altered expression and localization of nuclear envelope proteins in a prostate cancer cell system
Source: Mol Biol Rep. 2024 Aug 8;51(1):898. doi: 10.1007/s11033-024-09836-4 (PMC11310284; doi:10.1007/s11033-024-09836-4)
Supplement: Supplementary file 1 — Supplementary Material 1 [file 11033_2024_9836_MOESM1_ESM.pdf]

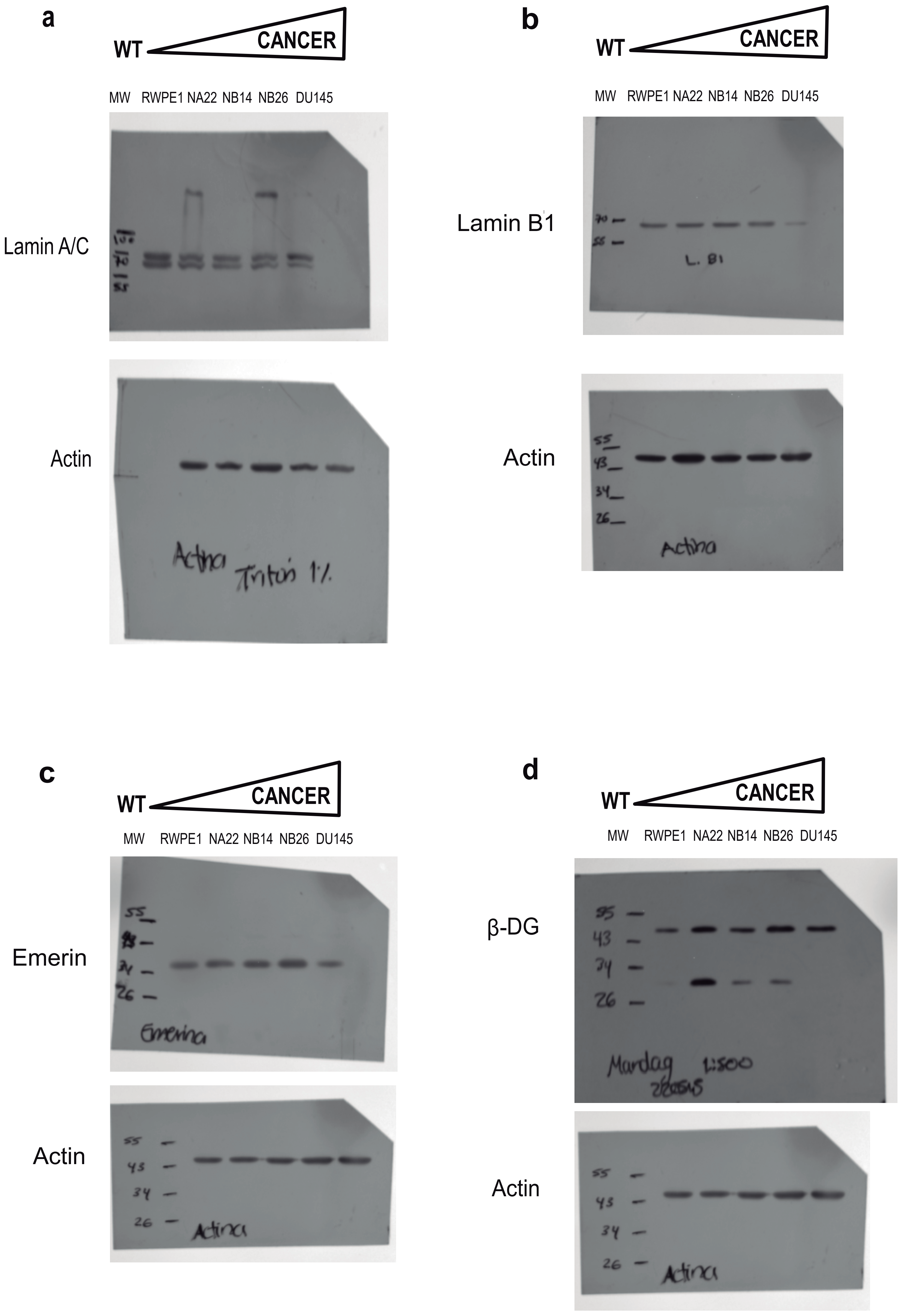
a
WT
CANCER
MW RWPE1 NA22 NB14 NB26 DU145
Lamin A/C
Actin
b
WT
CANCER
MW RWPE1 NA22 NB14 NB26 DU145
Lamin B1
Actin
c
WT
CANCER
MW RWPE1 NA22 NB14 NB26 DU145
Emerin
Actin
d
WT
CANCER
MW RWPE1 NA22 NB14 NB26 DU145
β-DG
Actin
